# Does postoperative pulmonary infection correlate with intestinal flora following gastric cancer surgery? — a nested case–control study

**DOI:** 10.3389/fmicb.2023.1267750

**Published:** 2023-11-01

**Authors:** Jie Yang, Yuhua He, Xi Liao, Jiankun Hu, Ka Li

**Affiliations:** ^1^Colorectal Cancer Center, West China Hospital, Sichuan University/West China School of Nursing, Sichuan University, Chengdu, China; ^2^Gastric Cancer Center, West China Hospital, Sichuan University, Chengdu, China; ^3^West China Hospital, Sichuan University/West China School of Nursing, Sichuan University, Chengdu, China

**Keywords:** pulmonary infection, gastric cancer, postoperative, intestinal flora, 16S rDNA, metagenomic analyses

## Abstract

**Introduction:**

The primary objective of this study was to investigate the potential correlation between gut microbes and postoperative pulmonary infection in gastric cancer patients. Additionally, we aimed to deduce the mechanism of differential functional genes in disease progression to gain a better understanding of the underlying pathophysiology.

**Methods:**

A nested case–control study design was utilized to enroll patients with gastric cancer scheduled for surgery at West China Hospital of Sichuan University. Patients were categorized into two groups, namely, the pulmonary infection group and the control group, based on the development of postoperative pulmonary infection. Both groups were subjected to identical perioperative management protocols. Fecal samples were collected 24 h postoperatively and upon pulmonary infection diagnosis, along with matched controls. The collected samples were subjected to 16S rDNA and metagenomic analyses, and clinical data and blood samples were obtained for further analysis.

**Results:**

A total of 180 fecal specimens were collected from 30 patients in both the pulmonary infection and control groups for 16S rDNA analysis, and 3 fecal samples from each group were selected for metagenomic analysis. The study revealed significant alterations in the functional genes of the intestinal microbiome in patients with postoperative pulmonary infection in gastric cancer, primarily involving Klebsiella, Enterobacter, Ruminococcus, and Collinsella. During postoperative pulmonary infection, gut flora and inflammatory factors were found to be associated with the lipopolysaccharide synthesis pathway and short-chain fatty acid (SCFA) synthesis pathway.

**Discussion:**

The study identified enriched populations of Klebsiella, Escherella, and intestinal bacteria during pulmonary infection following gastric cancer surgery. These bacteria were found to regulate the lipopolysaccharide synthesis pathway, contributing to the initiation and progression of pulmonary infections. Inflammation modulation in patients with postoperative pulmonary infection may be mediated by short-chain fatty acids. The study also revealed that SCFA synthesis pathways were disrupted, affecting inflammation-related immunosuppression pathways. By controlling and maintaining intestinal barrier function, SCFAs may potentially reduce the occurrence of pulmonary infections after gastric cancer surgery. These findings suggest that targeting the gut microbiome and SCFA synthesis pathways may be a promising approach for preventing postoperative pulmonary infections in gastric cancer patients.

## 1. Introduction

Gastric cancer (GC) is a prevalent malignant tumor of the digestive tract with a high incidence and mortality rate (GBD 2017 Stomach Cancer Collaborators, 2020). According to the International Agency for Research on Cancer (IARC) (Pizzato et al., 2022), there are approximately 1.089 million new cases of gastric cancer and 0.769 million deaths worldwide, ranking fifth among all malignant tumors and fourth in mortality. Surgical treatment is the most common and effective approach for treating gastric cancer (Kubota et al., 2014; Kanda et al., 2019). However, pulmonary infection is a frequent complication after GC surgery, with a morbidity rate of 10% ~ 25% (Lugg et al., 2017; Wanderer et al., 2021). Gastric cancer is most common in people over 55 years of age, who are physiologically deteriorated and less tolerant of surgical trauma (Chen et al., 2021). Besides that, gastric cancer surgery usually takes more than 3 h, the prolonged duration of general anesthesia increases the risk of pulmonary infection (Xiao et al., 2019). Furthermore, gastric cancer surgery is an upper abdominal surgery, which has a higher incidence of postoperative pulmonary infection than other surgeries such as lower abdominal, urologic and spinal surgeries (Xiao et al., 2019). Postoperative pulmonary infection significantly prolongs length of hospital stay and increases hospitalization costs, leading to a severe impact on postoperative rehabilitation and disease outcome (Kiuchi et al., 2016; Bui et al., 2020; Gertsen and Goense, 2020; Li et al., 2020).

The intestinal microbiome is closely associated with the occurrence and progression of pulmonary infection (Mjösberg and Rao, 2018). The concept of the pulmonary-intestinal axis suggests that there is bidirectional biological regulation between the intestinal tract and lungs. An animal study demonstrated that reducing the abundance and richness of Firmicutes and Bacteroidetes in the intestines significantly increased the risk of *Streptococcus pneumoniae* infection in experimental mice (Yoshida et al., 2018). A clinical study found that oral administration of Lactobacilli and Bifidobacteria resulted in a shorter duration of respiratory symptoms and lower percentages of antibiotics than the placebo group (Heinken et al., 2014). The composition and function of the digestive tract flora can affect the respiratory system through immune regulation (Zhuang et al., 2019; Zhang et al., 2020). The gut microbiome promotes the infiltration of immune T cells and the secretion of interleukin-1 (IL-1), improving pulmonary-related immune function (Youssef et al., 2018; Erawijantari and Mizutani, 2020). Additionally, the intestinal microecology promotes the aggregation of T-helper-cell 17 (Th-17) in the lungs and the phagocytic function of macrophages to improve pulmonary inflammation (Zafar and Saier, 2021). Some metabolites and proteins of the intestinal microbiome lead to negative infiltration of Treg cells in the lungs and inhibit the immune response, increasing the risk of pulmonary infection (Rivière et al., 2016). Moreover, the communication mechanism of the pulmonary-intestinal axis involves the direct migration of immune cells from the intestine to the respiratory tract through circulation, such as innate lymphoid cells 2 (ILC-2) and innate lymphoid cells 3 (ILC-3) (Mjösberg and Rao, 2018). The concept of the pulmonary-intestinal axis provides a new direction for exploring the prevention and treatment strategies of respiratory diseases from the perspective of intestinal microecology.

However, the mechanism of the intestinal microbiome in the progression of pulmonary infection after gastric cancer surgery remains unclear. The two objectives of this clinical study were (1) to identify alterations in the intestinal microbiome and pulmonary infection following gastric cancer surgery and (2) to identify key factors associated with pulmonary infection using 16S rDNA sequencing technology and metagenomic sequencing. The research findings may facilitate accurate monitoring and nursing of pulmonary infection after gastric cancer surgery.

## 2. Materials and methods

### 2.1. Study design

This study was approved by the Biomedical Ethics Committee of West China Hospital of Sichuan University [Trial No. 223 in 2021]. All study patients signed written informed consent. Use of convenience sampling method, patients diagnosed with gastric cancer who underwent surgical treatment at West China Hospital of Sichuan University from January 2021 to December 2021 were selected as study subjects. Patients without other metabolic or infectious diseases or distant metastasis of cancer cells were included, and all patients participated voluntarily. Exclusion criteria: ①Previous history of gastrointestinal surgery. ②Probiotics, microbial preparation, antibiotics, metformin, proton pump inhibitors, berberine and purgative agents were used in the last 3 months. ③ Intestinal inflammation, perforation, obstruction and severe systemic diseases were observed before the operation.

This nested case–control study enrolled all gastric cancer patients meeting the criteria in the study cohort. Patients with pulmonary infection were matched as the case group, and patients without pulmonary infection were selected from the same cohort as the control group. Relevant information such as age, gender, and disease of cases were matched. Pulmonary infection can be diagnosed according to the “Diagnostic Standards for Hospital Infection” of the People’s Republic of China, and if one of the following two conditions is met: (1) The patient has coughing, sticky phlegm, and moist rales in the lungs, with one of the following conditions: ① fever; ②X-ray (or CT) shows inflammatory infiltrating lesions in the lungs; ③An increase in white blood cell count and/or neutrophil ratio. (2) Acute infection secondary to stabilization of chronic airway disease with pathogenetic changes or X-ray chest radiograph (or CT) showing significant changes from the time of admission.

### 2.2. Perioperative management

The perioperative management for both groups of patients was the same, as follows. Preoperative preparation stage: (1) Patients without gastrointestinal dyskinesia fasted for 6 h and abstained from drinking for 2 h preoperatively. (2) nondiabetic patients were allowed to take carbohydrate drinks ≤400 mL for 2–3 h preoperatively. (3) No gastric tube was placed before surgery. (4) No intervention measures were given to patients before surgery, such as probiotics, antibiotics, proton pump inhibitors, berberine, or laxatives, which may affect the intestinal microbiota. Intraoperative treatment: (1) All surgeries are completed by the same medical team. (2) General anesthesia. (3) Patients undergone proximal subtotal gastrectomy, distal subtotal gastrectomy, or total gastrectomy. (4) The reconstruction method of the digestive tract was gastroesophagostomy, Billroth I, Billroth II, or Roux-en-Y. (5) Intraoperative prophylactic use of cefoxitin/cefmetazole 1 g, intravenous drip. Postoperative treatment: (1) No nasogastric tube was left in the patients after surgery. (2) On the first day after surgery, the patient began to consume clear fluid, attempted to consume semi liquid food on the second day. (3) Give daily intravenous drip of cefoxitin/cefmetazole, once every 8 h, 1 g each time. (4) No probiotics or other live bacterial preparations were used after surgery until discharge.

### 2.3. Sample collection

Fecal samples (3–5 g) were collected 24 h after surgery and pulmonary infection, and concurrent control samples were collected from the control group at the same time point. The specimens in the pulmonary infection group at 24 h postoperatively and at the time of occurrence of pulmonary infection were named pi-two, pi-three, respectively. The specimens in the control group at 24 h postoperatively and during the same period when pulmonary infection occurred in the case group were named con-two, con-three, respectively. Fecal samples were collected using the anal swab method, with the following steps: ①Clean the area around the anus with 70% alcohol. ②After moistening the sterile swab with physiological saline, insert it into the anus for 4–5 cm and gently rotate it. Take a sample from the anal recess, and obvious fecal marks can be seen on the swab. ③Insert the sampled swab into a sterile cryopreservation tube and store it in a − 80°C refrigerator. In the event of pulmonary infection, peripheral blood samples were collected from patients in the pulmonary infection group and the control group and submitted for testing white blood cell count and neutrophil ratioin a timely manner.

### 2.4. Microbiome analysis

Fecal specimen DNA extraction, PCR amplification, and library building were performed to construct species abundance profiles at the corresponding taxonomic level using 16S rDNA sequencing technology. Raw sequence processing, OTU classification, and species annotation were then performed. The samples used for metagenomic sequencing analysis were selected using MicroPITA analysis (Tickle et al., 2013). Metagenomic sequencing technology was used for genome assembly, gene prediction, nonredundant gene sets, metagenome analysis, and flow chart (including dilution curve drawing of core-pan genes, gene number analysis between groups, differential species analysis of metagenes, alignment analysis of egg NOG database, and KEGG database alignment analysis). General and clinical data were also collected to analyze the correlation between inflammatory indicators and intestinal flora.

### 2.5. Statistical analysis

The study was conducted using the R software package. General and clinical data were analyzed using two independent sample t tests, chi-square tests, or Fisher’s exact probability tests. Species richness and distribution evenness were evaluated by calculating the Chao1 index, Shannon index, and Simpson index for α diversity analysis. The community similarity and difference of different groups were evaluated by β diversity analysis. The significance of the sample community distribution between groups was evaluated by NMDS analysis based on the Bray–Curtis distance matrix algorithm and the difference significance *p* value in Adonis analysis (also based on the Bray–Curtis distance matrix algorithm). The structure of the intestinal flora at different taxonomic levels was expressed by a boxplot diagram. Postpoly NMDS mapping was performed using the partitioning around medoids method according to the Jensen–Shannon divergence distance. Differences in KO between the two groups were compared using the Wilcoxon rank-sum test. Differential pathways and differential modules were calculated using the Z score. The correlation of Map/Module to patient general and clinical data and inflammatory indicators was calculated using Spearman correlation analysis.

## 3. Results

A total of 120 stool samples were collected, with 30 patients each from the pulmonary infection group and the control group. No participants were lost during the experimental period of the study. There were no significant differences identified between the groups in terms of age, sex, preoperative comorbidities, preoperative neoadjuvant chemotherapy, surgical procedures, and intraoperative blood loss, et al (Table 1).

**Table 1:** Comparison of demographic characteristics between the pulmonary infection group and the control group.

Parameter	Pulmonary infection group (*n* = 30)	Control group (*n* = 30)	*p*
Age, years, mean ± SD	64.87 ± 12.02	65.33 ± 11.40	0.878
Nation (the Han nationality/Others), *n*	30/0	27/3	0.237
Sex (Male/Female), *n*	19/11	17/13	0.598
Preoperative comorbidity (No/Yes), *n*	0/30	5/25	0.052
Preoperative neoadjuvant chemotherapy (Yes/No), *n*	15/15	13/17	0.605
Surgical approach (Laparoscopy/Open), *n*	7/23	12/18	0.165
Digestive tract reconstruction (Gastroesophagostomy/Billroth I/Billroth II /Roux-en-Y), *n*	7/6/4/13	9/6/5/10	0.917
Resection range (Proximal gastrectomy/Distal subtotal gastrectomy/Total gastrectomy), *n*	7/10/13	9/11/10	0.709
Intraoperative blood loss, ml, mean ± SD	60.00 ± 20.42	45.50 ± 22.87	0.094
Surgical duration, hours, mean ± SD	4.68 ± 0.81	4.76 ± 0.79	0.690
Body mass index, kg/m^2^, mean ± SD	24.08 ± 2.36	22.83 ± 2.36	0.051
ASA score (1/2/3), *n*	2/11/17	0/16/14	0.208

Statistical test performed: two-sample *t*-test for numerical data; Chi-square test or Fisher’s exact test for categorical data; *n*, number of participants.

### 3.1. 16S rDNA sequencing

#### 3.1.1. Comparison of α diversity and β diversity between the pulmonary infection group and the control group at 24 h post postoperatively

The Chao1 index, Shannon index, and Simpson index were used to reflect the α diversity. The Wilcoxon rank sum test results showed no significant difference in the Chao1 index, Shannon index, and Simpson index between the pulmonary infection group and the control group at 24 h post postoperatively (all *p* > 0.05).

**Figure 1:**
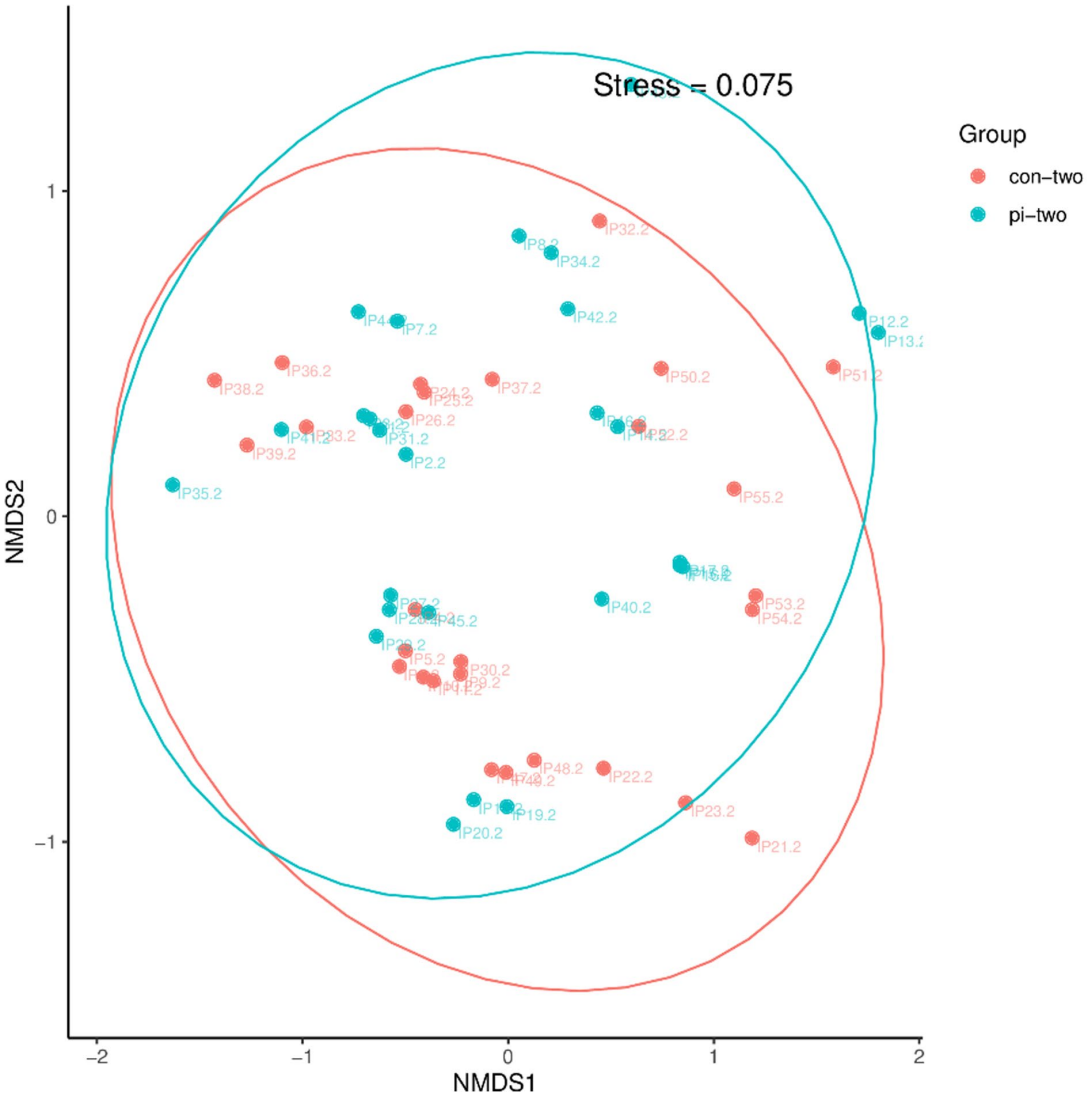
Analysis of intestinal flora β diversity in the pulmonary infection group compared with the control group at 24 h postoperatively.

The β diversity of the intestinal microbiota between the pulmonary infection group and the control group at 24 h post postoperatively was analyzed using the NMDS algorithm of weighted UniFrac, as shown in Figure 1. The Adonis statistical analysis with the results of the Bray–Curtis distance algorithm found no significant difference between the β diversity of the two groups (*p* = 0.208). Therefore, further comparison of gut microbiota structure at different taxonomic levels will not be conducted.

#### 3.1.2. Comparison of α diversity, β diversity and the structure of the intestinal flora at different taxonomic levels in the pulmonary infection group at the time of the occurrence of pulmonary infections and in the concurrent control group

The Chao1 index, Shannon index, and Simpson index were used to reflect the α diversity of patients in the pulmonary infection group and the contemporary control group. The Wilcoxon rank sum test results showed no significant difference in the Chao1 index, Shannon index, and Simpson index between the two groups (all *p* > 0.05).

**Figure 2:**
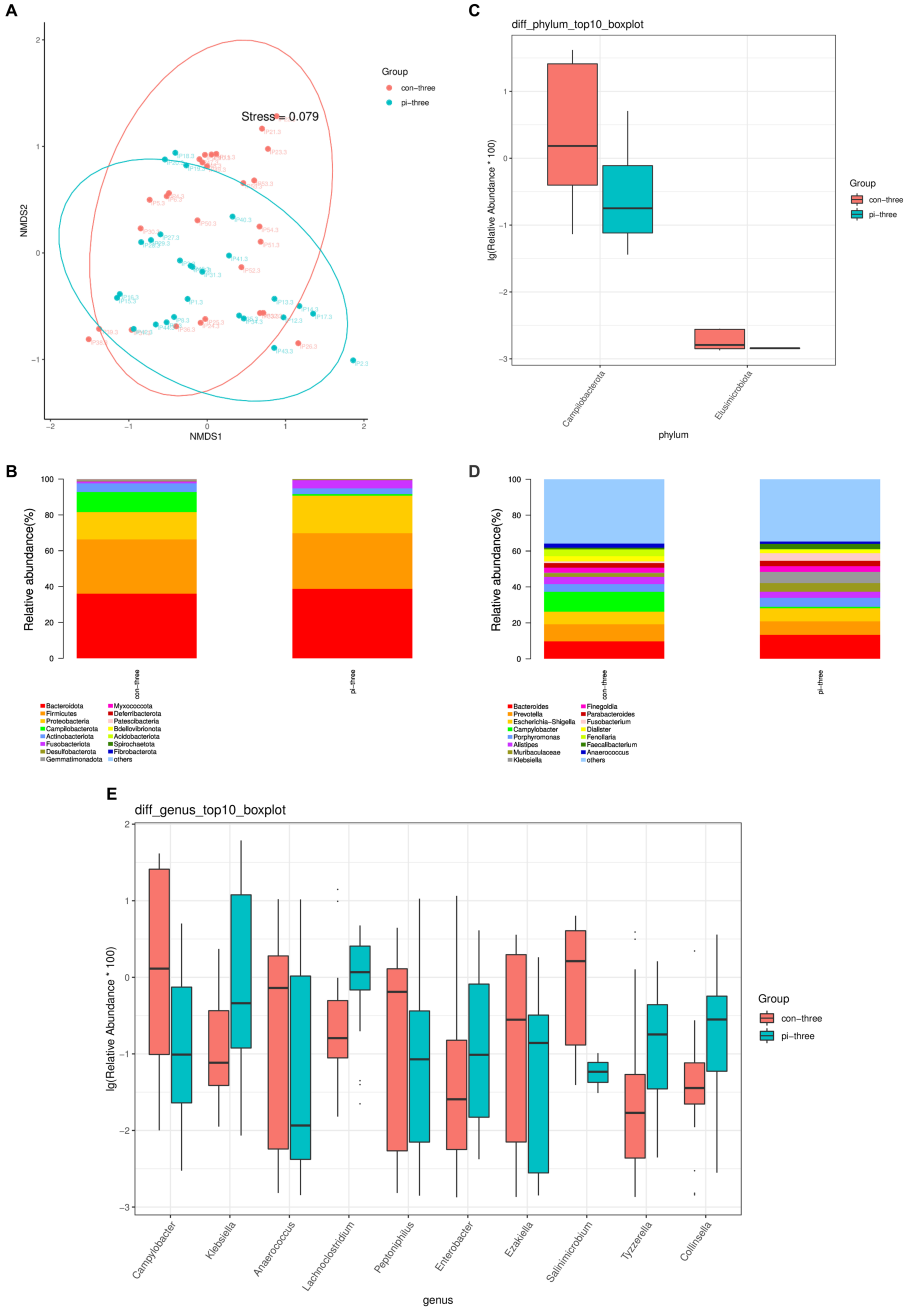
**(A)** Analysis of intestinal flora β diversity, **(B)** Top 15 species at the phylum level, **(C)** Differential flora at the phylum level, **(D)** Top 15 species in relative abundance at the genus level, **(E)** Top 10 different flora in relative abundance at the genus level in the pulmonary infection group compared with the contemporary control group when pulmonary infection occurred.

The β diversity of the intestinal microbiota in the pulmonary infection group and the contemporary control group was analyzed using the NMDS algorithm of weighted UniFrac, as shown in Figure 2A. The Adonis statistical analysis with the results of the Bray–Curtis distance algorithm found a significant difference between the β diversity of the pulmonary infection group and the contemporary control group (*p* = 0.045). The Adonis statistics showed that the R^2^ value of the grouping factors was 0.042.

The top 15 relative abundances of patients at the phylum level in the pulmonary infection group and the contemporary control group are shown in Figure 2B. According to the Wilcoxon rank sum test, there were two different bacteria in the intestinal tract in the pulmonary infection group (all *p* < 0.05), as shown in Figure 2C. Campilobacterota and Elusimicrobiota were enriched in the control group and had low relative abundance in the pulmonary infection group (*p* < 0.05).

The top 15 relative abundances of patients at the genus level of the pulmonary infection group and the contemporary control group are shown in Figure 2D. According to the Wilcoxon rank sum test, there were 75 different bacteria in the gut of the pulmonary infection group and the contemporary control group (*p* < 0.05). The top 10 differentially abundant species at the genus level of pulmonary infection in the pulmonary infection group and the contemporary control group are shown in Figure 2E: Campylobacter, Klebsiella, Anaerococcus, Lachnoclostridium, Peptoniphilus, Enterobacter, and Ezak, Salinimicrobium, Tyzzerella, Collinsella.

#### 3.1.3. Comparison of α diversity, β diversity and the structure of the intestinal flora at different taxonomic levels at 24 h postoperatively and at the time of pulmonary infection in the pulmonary infection group

The Chao1 index, Shannon index, and Simpson index were used to reflect the α diversity of the intestinal flora. The Wilcoxon rank sum test showed no significant difference between the Chao1 index, Shannon index, and Simpson index at 24 h postoperatively and at the time of pulmonary infection in the pulmonary infection group (*p* > 0.05).

**Figure 3:**
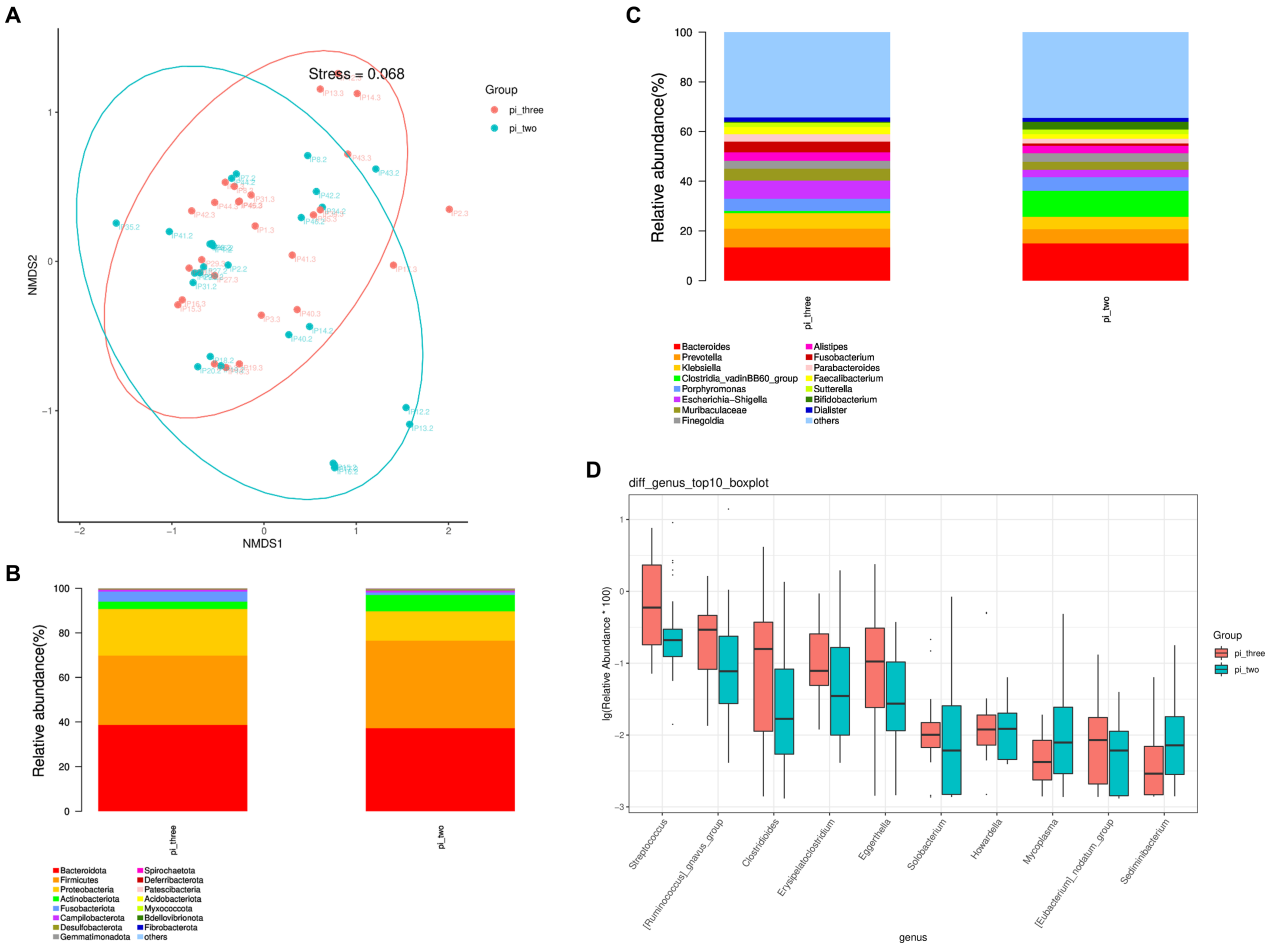
**(A)** Analysis of intestinal flora β diversity, **(B)** Top 15 species in relative abundance at the phylum level, **(C)** Top 15 species in relative abundance at the genus level, **(D)** Top 10 different flora in relative abundance at the genus level at 24 h postoperatively and at the time of the occurrence of the pulmonary infection in the pulmonary infection group.

The β diversity of the pulmonary infection group at 24 h postoperatively and at the time of the occurrence of the pulmonary infection was analyzed using the NMDS algorithm of weighted UniFrac, as shown in Figure 3A. The Adonis statistical analysis with the results of the Bray–Curtis distance algorithm found a significant β diversity difference between 24 h postoperatively and at the time of the occurrence of the pulmonary infection (*p* = 0.001), and the R^2^ value of the grouped factors was 0.108. The top 15 relative abundances of species at the phylum level at 24 h postoperatively and at the time of the occurrence of the pulmonary infection in the pulmonary infection group is shown in Figure 3B. According to the Wilcoxon rank sum test, there were no differences in the pulmonary infection group at 24 h and at the time of pulmonary infection at the phylum level (*p* > 0.05).

The top 15 relative abundances of species at the genus level at 24 h postoperatively and at the time of the occurrence of the pulmonary infection in the pulmonary infection group is shown in Figure 3C. A total of 21 differential flora were present in the pulmonary infection group at 24 h postoperatively and at the time of the occurrence of the pulmonary infection (*p* < 0.05). Specifically, the top 10 differentially abundant flora are shown in Figure 3D. The abundance of Mycoplasma and Sediminibacterium were significantly reduced at the time of pulmonary infection compared to 24 h postoperatively (*p* < 0.05), while Streptococcus, Ruminococcus, Clostridioides, Erysipelatoclostridium, Eggerthella, Solobacterium, Howardella, and Eubacterium were significantly increased (*p* < 0.05).

### 3.2. Metagenomic sequencing

#### 3.2.1. NCBI-NR analysis of the database alignment

**Figure 4:**
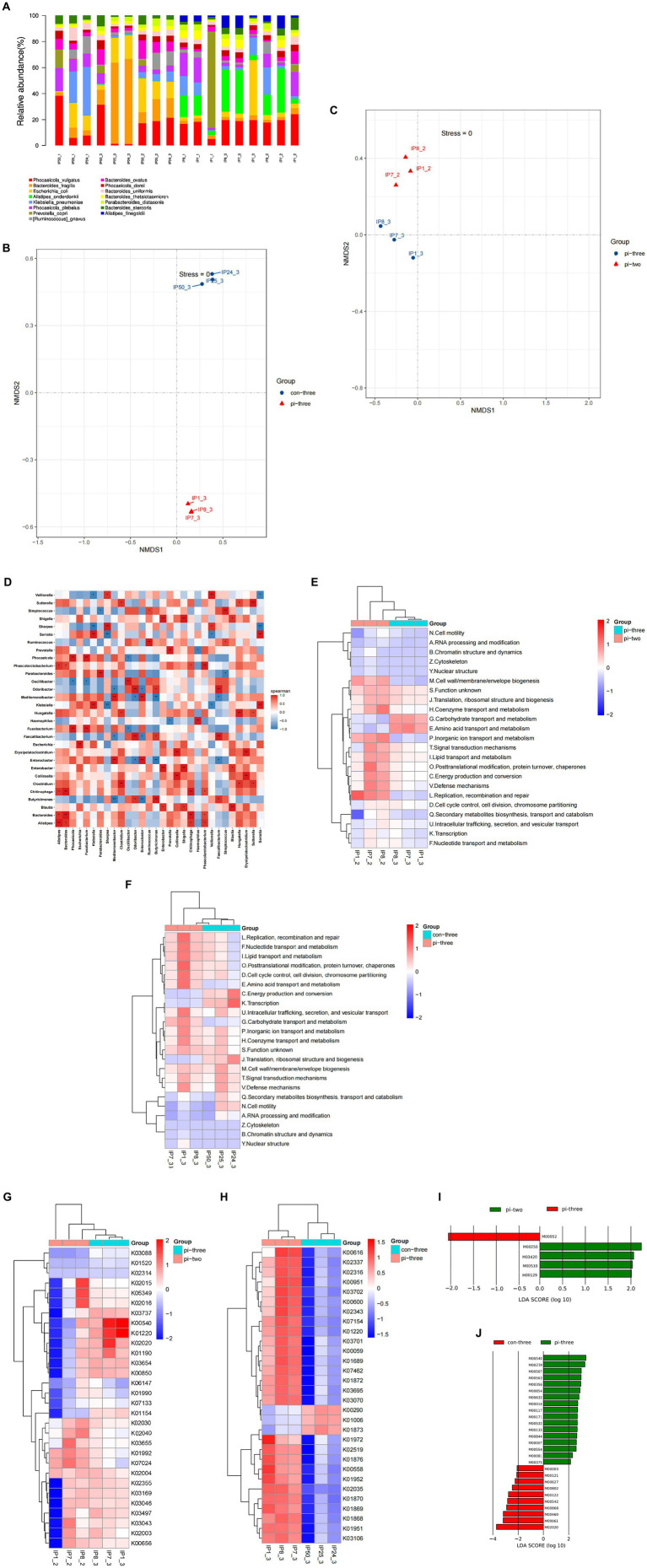
**(A)** Each sample is annotated to a bar chart of relative abundance of species, **(B)** NMDS analysis of intestinal microorganisms for pulmonary infection group compared with the contemporary control group when pulmonary infection occurred, **(C)** NMDS analysis of intestinal microorganisms at 24 h after surgery compared with when pulmonary infection occurred in pulmonary infection group, **(D)** heatmap of correlations between different species, **(E)** differential eggNOG analysis at 24 h after surgery compared with when pulmonary infection occurred in pulmonary infection group, **(F)** differential eggNOG analysis for pulmonary infection group compared with the contemporary control group when pulmonary infection occurred, **(G)** differential KO analysis at 24 h after surgery compared with when pulmonary infection occurred in pulmonary infection group, **(H)** differential KO analysis for pulmonary infection group compared with the contemporary control group when pulmonary infection occurred, **(I)** differential expression of metabolic pathways at 24 h after surgery compared with when pulmonary infection occurred in pulmonary infection group, **(J)** differential expression of metabolic pathways for pulmonary infection group compared with the contemporary control group when pulmonary infection occurred.

Species annotations were obtained by alignment with the taxonomic information database corresponding to the NCBI-NR database. The results showed that among the genes enriched in pulmonary infection, 535 genes were annotated at the species level, with most of them distributed in Klebsiella (263), Prevotella (79), Escherichia (121), and Ruminococcus (52). Among the genes enriched at 24 h after surgery in the pulmonary infection group, 56 were annotated to the species level, of which 53.25% belonged to Phocaeicola (27) and *Bacteroides fragilis* (19). Among the genes enriched in the contemporary control patients, 23 genes were annotated at the species level, of which 57.34% were distributed in Phocaeicola plebeius (Figure 4A).

NMDS analysis was applied to represent the species information. Each dot in the figure represents a sample, and the same color represents the same grouping. The distance between points reflects the degree of difference between different samples, with the closer the sample of the same group and the more obvious the distance from other groups. The results showed a significant difference in the species abundance of the intestinal microecology between the pulmonary infection group and the contemporary control group (*p* < 0.05), as shown in Figure 4B. The species abundance of gut microorganisms between the pulmonary infection group and the group at 24 h after surgery was also significantly different (*p* < 0.05; Figure 4C), which was consistent with 16S rDNA sequencing.

The top 30 species in total abundance at the genus level were selected, and a heatmap map of related differential species was drawn. The horizontal and vertical coordinates represent the corresponding species, with a negative correlation in blue and a positive correlation in red. As shown in Figure 4D, there was a positive correlation between the bacteria enriched in the pulmonary infection group and a negative correlation between the pulmonary infection group and the other two groups (*p* < 0.05).

#### 3.2.2. Egg NOG analysis of the database alignment

The comparison of nonredundant genes with the egg NOG database showed that orthologous groups (OGs) added 774, 1,298, and 513 OGs in the pulmonary infection group 24 h after surgery, at the time of infection, and in the contemporary control group, respectively. Compared with 24 h after surgery, the functional gene clusters that increased when pulmonary infection occurred were mainly distributed in [G] (Carbohydrate transport and metabolism), [E] (Amino acid transport and metabolism) (*p* < 0.05), as shown in Figure 4E. When pulmonary infections occurred in the case group, the functional gene clusters in the control group during the same period were predominantly distributed in [J](Translation,ribosomal structure and biogenesis), [C] (Energy production and conversion), [K](Transcription), as shown in Figure 4F.

#### 3.2.3. Alignment and analysis of the KEGG database

The intestinal microbial function genes were involved in many biological metabolic pathways. In particular, the functional genes with the largest number of intestinal microbes were carbohydrate metabolism, accounting for 18.76%, and amino acid metabolism, accounting for 13.27%. Compared with 24 h after surgery of the pulmonary infection group, the relative abundance of gut microbial genome annotation to the KEGG module in the event of pulmonary infection was significantly increased in lipopolysaccharide biosynthesis (*K00540*), signal transduction system (*K02020*), heterogeneous biomass degradation (*K01220*). However, the KEGG module abundance of cell motility chemotaxis (*K02030*), flagellar assembly (*K02040*) was significantly reduced (all *p* < 0.05), as shown in Figure 4G.

The gut microbial genome was annotated to KEGG when pulmonary infection occurred compared with the contemporary control group. The significant increase in module relative abundance was involved in xenobiotic degradation (*K01220*) and ornithine (*K00616*). Significant reduction in KEGG module abundance of isoleucine organisms synthesis (*K00290*) was observed (all *p* < 0.05), as shown in Figure 4H.

#### 3.2.4. Metabolic pathway analysis of differential expression

As shown in Figure 4I, compared with 24 h after surgery, metabolic pathways (*M00250*) and RNA polymerases that are closely related to the alanine and glutamate metabolic pathways (*M03420*) and the abundance of metabolic pathways associated with the biosynthesis of peptidoglycan (*M00510*) were also significantly reduced (all *p* < 0.05).

As shown in Figure 4J, the associated metabolic pathway abundance increased significantly with lipopolysaccharide (LPS) biosynthesis (*M00540*) and purine nucleotide synthesis (*M00230*) involved in pulmonary infection compared with concurrent control patients (all *p* < 0.05). In contrast, the metabolic pathways associated with short-chain fatty acid (SCFA) formation (*M02020*), isoleucine biosynthesis (*M00460*), and fatty acid biosynthesis and oxidation (*M00061*) were significantly reduced in the above two groups (all *p* < 0.05).

#### 3.2.5. Correlation between clinical data, inflammatory indicators, intestinal flora and pathway

The intestinal flora, clinical data, inflammatory indicators, funtional and metabolic pathways of the pulmonary infection group were compared with those of the contemporary control group, including age, sex, complications, BMI, remodeling pattern of the digestive tract, and inflammatory index. There was a positive correlation between Klebsiella, lipopolysaccharide biosynthesis and the inflammation index, including white blood cell count and neutrophil ratio (all *p* < 0.05). However, the metabolic pathways associated with carbohydrate biosynthesis, isoleucine biosynthesis, and short-chain fatty acid biosynthesis were inversely related to leukocyte count and neutrophil ratio (all *p* < 0.05).

## 4. Discussion

### 4.1. The correlation between postoperative pulmonary infection and intestinal microbiota structure in GCs

In this study, we compared the α diversity of the intestinal flora between the pulmonary infection group and the control group at 24 h postoperatively and at the time of occurrence of pulmonary infection, respectively, and no significant differences were found. Similarly, there was no difference in alpha diversity in the pulmonary infection group at the time of occurrence of pulmonary infection compared to 24 h postoperatively. This suggests that the species, i.e., the total number, of intestinal flora does not change significantly during pulmonary infection. A study by Youssef et al. (2018) also confirmed that the overall number of intestinal flora is usually relatively constant, subject to factors such as the disease itself, diet, and therapeutic interventions, although certain intestinal flora may undergo transient changes in relative abundance during the course of disease onset and progression. So, which flora undergo changes in their relative abundance during the development of pulmonary infections? Based on this, we further analyzed the β diversity of the intestinal flora to clarify whether there was a significant change in the relative abundance of one and several conditionally pathogenic bacteria at the onset of pulmonary infection.

At 24 h postoperatively, there was no statistically significant difference in gut flora β diversity between the pulmonary infection group and the control group. However, in the event of pulmonary infection is, differences in β diversity were found between the pulmonary infection group and the contemporary control group. Further comparisons of the structure of the intestinal flora revealed that, when compared with the contemporary control group, Klebsiella, Enterobacter, and Collinsella were the enriched bacteria in the pulmonary infection group. Klebsiella can cause pulmonary infection by expressing a variety of pathogenic factors, including multiple adhesins, capsular polysaccharides, siderophores, and LPS. Studies have confirmed that the capsular polysaccharide of *Klebsiella pneumoniae* inhibits dendritic cell maturation, thereby reducing the production of cytokines such as IL-12 and TNF-α to regulate the inflammatory response (Russo and Marr, 2019). Enterobacter belongs to the family of Enterobacteriaceae, which is a risk factor for hospital-acquired and ventilator-associated gram-negative bacterial pneumonia (Thibeault et al., 2021). Collinsella is a very abundant member of the phylum Actinobacteria. Pro-inflammatory properties of its bacterial cell wall have been reported (Mena-Vázquez et al., 2020).

Furthermore, we found that the abundance of Mycoplasma was significantly reduced at the time of pulmonary infection, while the abundance of Streptococcus, Ruminococcus, and Clostridioides was significantly increased in the pulmonary infection group compared with 24 h after surgery (all *p* < 0.05). Streptococcus produce virulence factors by secretion of podocarboxylic polysaccharides, and enhance their invasiveness and virulence by promoting the body’s production of neuraminidase hemolysin, phospholipid wall acid, etc. (Castro-Leyva et al., 2022). Group A Streptococcus and *Streptococcus pneumoniae*, can cause a variety of purulent inflammation and hypersensitivity reactive diseases (Kanwal and Vaitla, 2023). Ruminococcus synthesizes and secretes complex polysaccharides with glucose side chains as well as a rhamnose backbone, which in turn induces an increase in inflammatory cytokines (Pal et al., 2021). Studies have shown that the increased relative abundance of Ruminococcus_gnavus in patients with Crohn’s disease, active phase inflammatory bowel disease, and irritable bowel syndrome is associated with severe symptoms (Sundin et al., 2015; Henke et al., 2019). In addition, Clostridioides is one of the most common nosocomial infections (Oliveira et al., 2020) and colonizes the gut, especially after the destruction of the normal gut flora.

There were some interesting findings. Among the flora enriched in the pulmonary infection group described above, Enterobacter, Collinsella, Ruminalococcus, and Clostridioides difficile are the flora that would have been predominantly colonized in the gut. The relative abundance of these flora increased significantly in the presence of pulmonary infections. It is possible that respiratory infections cause disturbances in the structure of the intestinal flora. Moreover, interventions such as postoperative supine or semirecumbent position of the patient and indwelling diets promote gastroesophageal reflux, which allows these bacteria to migrate up to the oral cavity and serve as reservoirs for possible pathogens of pulmonary infections. In contrast, Klebsiella and Streptococcus which are flora that primarily reside in the human nasopharyngeal-oral region, appeared in the intestinal tract with increased abundance in the occurrence of pulmonary infections, further suggesting the existence of a bi-directional pathway between the lungs and the intestinal tract, which is consistent with the idea of a pulmonary-intestinal axis. Some researchers have put forward the theory that lung flora and gut flora may be exchanged with the help of fluids in the lymph, and even that gut flora can colonize directly in the respiratory tract and thus affect lung health (Chakradhar, 2017). Although the underlying mechanisms may be multifaceted and remain to be further investigated.

### 4.2. The correlation between postoperative pulmonary infection, functional genes and metabolic pathways in GCs

The structure of the patient’s intestinal flora changed significantly during pulmonary infections. In order to better understand the mechanism of intestinal flora in the development of postoperative pulmonary infection after gastric cancer, the functional genes and metabolic pathways of differential intestinal microorganisms were further analyzed by macrogenomic sequencing.

The results showed that most of the genes enriched after pulmonary infection were distributed in Klebsiella, Prevotella, Escherichia, and Clostridium, while the gene abundance of Bacteroides was significantly reduced. Klebsiella is an important pathogen of respiratory tract infections, and its capsule polysacharides (CPS) will inhibit the maturation of dendritic cells, thus reducing the production of cytokines, such as IL-12 and TNF-α, which play an important role in the regulation of inflammatory responses in the body (Russo and Marr, 2019). Wang found that after reducing the richness and number of intestinal flora Bacteroides, experimental mice were more likely to develop pneumonia (Wang et al., 2014). Prevotella is often associated with systemic diseases and local infection, which possibly enhances Th17-mediated mucosal inflammation (Renyong et al., 2010). In addition, Prevotella effectively promotes the high expression of IL-8 through LPS on the surface, increasing the inflammatory response.

In this study, significant differences in gene function were observed among the three groups by comparison with the egg NOG functional gene database. The related functional genes were found to be more active in pulmonary infection after gastric cancer surgery, such as LPS biosynthesis (*K00540*), signal transduction system (*K02020*), and foreign biomass degradation (*K01220*). Available studies reported that LPS biosynthesis (*K00540*) is widely distributed in the Gram-negative bacteria, such as Fusobacteria and Proteobacteria. Lipopolysaccharide is a component of the outer wall of the cell wall of these Gram-negative bacteria. In this study, Enterobacter and Clostridioides, which were enriched in pulmonary infection group occurring after gastric cancer surgery, belonged to the phylum Proteobacteria and Fusobacteria, respectively. The enrichment of these Gram-negative bacteria in pulmonary infection occurring after gastric cancer surgery caused an increase in functional genes related to LPS synthesis, which resulted in an upregulation of lipopolysaccharide synthesis in patients with pulmonary infection occurring after gastric cancer surgery. The NF-kB pathway is activated by LPS-binding proteins, which play a key role in inflammation and immunity processes. In a study by Russa, LPS was found to be associated with T-cell activation and elevated pro-inflammatory responses, leading to a “cytokine storm” (Russo and Marr, 2019). Dysbiosis of intestinal flora promotes the entry of lipopolysaccharides into the portal circulation, which further stimulates Kupffer cells in the periportal area of the liver, leading to activation of the NF-kB pathway and secretion of TNF-α and TNF-β, resulting in hepatic and even systemic inflammation (Zhuang et al., 2019). In addition to the direct effects of LPS on the body’s inflammatory response through immune activation, high plasma LPS levels have been shown to increase intestinal permeability (Zhuang et al., 2019). And increased intestinal permeability will lead to bacterial translocation into the body’s circulation, triggering a systemic inflammatory response. A study examined the characterization of microbial levels in lung lavage fluid from patients with acute respiratory distress syndrome (ARDS) and showed that Bacteroides was detected in the lung lavage fluid of 33% of patients with ARDS, whereas it was not detected in the lungs of the healthy population. It is evident that Bacteroides enriched in the intestine may migrate through the highly permeable intestinal wall to the lungs of patients with severe lung injury (Dickson et al., 2016). Therefore, we hypothesized that high levels of LPS in the intestines of patients who developed pulmonary infection after gastric cancer surgery may also contribute to the migration of pathogenic bacteria, such as Klebsiella, into the lungs of patients by increasing intestinal permeability. Furthermore, the functional gene related to the signal transduction system (*K02020*) and foreign biomass degradation (*K01220*) increased in pulmonary infection (Zou et al., 2019), which were prevalent in Klebsiella, Escherichia, and Enterobacter. Abundant signal transduction and foreign biomass degradation systems enable these colonies genera to sense and respond to a wide range of stresses and toxic substances present in the environment.

In comparison with the KEGG metabolic pathway database, metabolic pathways associated with carbohydrate degradation were significantly reduced in the pulmonary infection group when pulmonary infection occurred, suggesting that postoperative pulmonary infection in gastric cancer impaired the ability of intestinal microorganisms to degrade carbohydrates. The metabolic pathways for short chain fatty acid (SCFA) production, isoleucine synthesis, and acetyl coenzyme synthesis were significantly reduced in abundance, suggesting that the synthesis of short chain fatty acids and isoleucine may be reduced in the intestines of patients with pulmonary infection occurring after gastric cancer surgery. SCFA are produced by bacteria in the gut when fermenting dietary fiber and are beneficial metabolites. SCFA are mainly derived from the metabolism of *Anaplasma phagocytophilum*, Lactobacillus, Streptococcus, etc., and include acetate, propionate, and butyrate. In the present study, the abundance of Bacteroidetes phylum was reduced when pulmonary infection occurred after gastric cancer surgery, which may be the main reason affecting the reduced synthesis of SCFA. Mucosal immunity is regulated by SCFAs by promoting B-cell development, differentiation and expansion of Tregs, inflammasome activation and IL-18 production (Lavelle and Sokol, 2020). Moreover, butyrate, the metabolite of SCFAs, plays an important role in reducing systemic inflammation by activating G protein-coupled receptors and inhibiting the NF-kB signaling pathway and maintaining the intestinal barrier to prevent the invasion of intestinal endotoxins and bacteria (Lavelle and Sokol, 2020). Therefore, downregulation of metabolic pathways associated with SCFA synthesis attenuates the ability of intestinal microbes to resist inflammatory responses. SCFA can also indirectly affect the intestinal mucosal barrier by activating short-chain fatty acid receptors on immune cells. Our study also found that the reduced abundance of SCFAs was related to immunity and the inflammatory response in patients with pulmonary infection after GC surgery. In addition, the biosynthetic pathway of isoleucine in this study also decreased in the abovementioned patients. Isoeucine triggers elevated expression of protective polypeptides, such as β-defensins, which regulate innate immunity as well as adaptive immune responses. SCFA can also indirectly affect the intestinal mucosal barrier by activating short-chain fatty acid receptors on immune cells (Xia et al., 2021). We therefore hypothesized that the gradual transition of the diet from fasting to a liquid diet in the early postoperative period in patients with gastric cancer and the low fiber content of the dietary structure may also lead to a decrease in the synthesis of SCFA. The modulation of the inflammatory response in patients with pulmonary infection occurring after gastric cancer surgery may be the result of disruption of pathways related to SCFA synthesis caused by a decrease in the abundance of SCFA-producing bacteria, which, in turn, also affects the important roles of these pathways in inflammation-associated immunosuppression and maintenance of intestinal barrier function. In addition, the diet of gastric cancer patients in the early post-surgical period gradually transitions from fasting to a liquid diet with a diet structure that contains less dietary fiber. Since the amount and type of dietary fiber is a major determinant of the production of the metabolite SCFA by the intestinal flora, this can also lead to a decrease in the synthesis of SCFA. These can lead to prolonged inflammation.

The results of the correlation analysis showed that there was a positive correlation between Klebsiella, lipopolysaccharide biosynthesis and the inflammation index, including white blood cell count and neutrophil ratio (all *p* < 0.05). However, the metabolic pathways associated with carbohydrate biosynthesis, isoleucine biosynthesis, and short-chain fatty acid biosynthesis were inversely related to leukocyte count and neutrophil ratio (all *p* < 0.05). These results once again validate the promotional role of LPS in the development of postoperative pulmonary infection after gastric cancer surgery and suggest that downregulation of metabolic pathways associated with SCFA synthesis attenuates the ability of gut microbes to resist inflammatory responses.

## 5. Conclusion and prospects

In this study, we found that postoperative pulmonary infection after gastric cancer was associated with the enrichment of Klebsiell, Enterobacter, rumenococcus Ruminococcus, and Collinsella in the intestine. Klebsiella and Enterobacterenriched in the intestinal tract in the case of postoperative pulmonary infection after gastric cancer surgery may play an important role in the occurrence and development of pulmonary infection through the regulation of the pathway of lipopolysaccharide synthesis. Pulmonary infection occurring after gastric cancer surgery may be the result of disruption of pathways related to SCFA synthesis in patients, which in turn affects the important role of these pathways in inflammation-associated immunosuppression and maintenance of intestinal barrier function. These results suggest that (1) oral cleansing is necessary to prevent pulmonary infection due to colonization of the upper respiratory tract with pathogenic bacteria. (2) Considering that there may be a bidirectional pathway between the lungs and the intestines, the monitoring of the intestinal flora of postoperative patients should be strengthened to recognize flora changes. In addition, intestinal homeostasis can be promoted by supplementing beneficial bacteria and their metabolites to indirectly promote lung health. (3) Give postoperative gastric cancer patients reasonable dietary instructions, such as appropriate dietary fiber supplementation preparations, thereby increasing SCFA, which will facilitate inflammation suppression.

This study also has certain shortcomings. First, although this study confirmed that the down-regulation of metabolic pathways related to SCFA synthesis may attenuate the ability of intestinal microorganisms to resist inflammatory responses, it is insufficient to indicate exactly what metabolites are playing a key role because metabolites were not detected and analyzed in this study. Furthermore, the results of the study were not verified in animal experiments, so it is necessary to further validate the role of differential flora in the regulation of pulmonary infection by constructing an animal model of postoperative pulmonary infection after gastric cancer in the future.

## Data availability statement

The original contributions presented in the study are included in the article/supplementary material, further inquiries can be directed to the corresponding authors.

## Ethics statement

The studies involving humans were approved by Biomedical Ethics Committee of West China Hospital of Sichuan University. The studies were conducted in accordance with the local legislation and institutional requirements. The participants provided their written informed consent to participate in this study.

## Author contributions

JY: Funding acquisition, Project administration, Writing – review & editing. YH: Data curation, Writing – original draft. XL: Writing – review & editing. JH: Methodology, Writing – review & editing. KL: Supervision, Writing – review & editing.

## Abbreviations

GC: Gastric cancer
IARC: International Agency for Research on Cancer
IL-1: Interleukin-1
ILC-2: Lymphoid cells 2
ILC-3: Lymphoid cells 3
LPS: Lipopolysaccharide
OGs: Orthologous groups
SCFAs: Short-chain fatty acids
Th-17: T-helper-cell 17
